# Ferroptosis: the vulnerability within a cancer monster

**DOI:** 10.1172/JCI170027

**Published:** 2023-05-15

**Authors:** Wanqing Xie, Shivani Agarwal, Jindan Yu

**Affiliations:** 1 Department of Urology, Emory University School of Medicine, Atlanta, Georgia, USA.; 2 Division of Hematology/Oncology, Department of Medicine, Northwestern University, Chicago, Illinois, USA.; 3 Department of Human Genetics and; 4 Winship Cancer Institute, Emory University School of Medicine, Atlanta, Georgia, USA.

## Abstract

Treatment-resistant cancer, such as neuroendocrine prostate cancer (NEPC), is a lethal disease with limited therapeutic options. *RB1* is a tumor suppressor gene that is lost in a majority of NEPC tumors. In this issue of the *JCI*, Wang and colleagues examined how RB1 loss may sensitize cancer cells to ferroptosis inducers through elevation of ACSL4, a key enzyme that promotes lipid peroxidation and triggers ferroptosis. We discuss a high potential of RB1-deficient cells to undergo ferroptosis due to the elevation of ACSL4. This is normally kept in check by abundant expression of GPX4, an antioxidant enzyme, in cancer cells. This balance, however, is tilted by GPX4 inhibitors, leading to massive ferroptosis. We highlight possible therapeutic strategies that exploit this inherent vulnerability for targeting *RB1*-deficient, treatment-resistant cancer.

## Emerging roles of ferroptosis in cancer and treatment resistance

**Figure 1:**
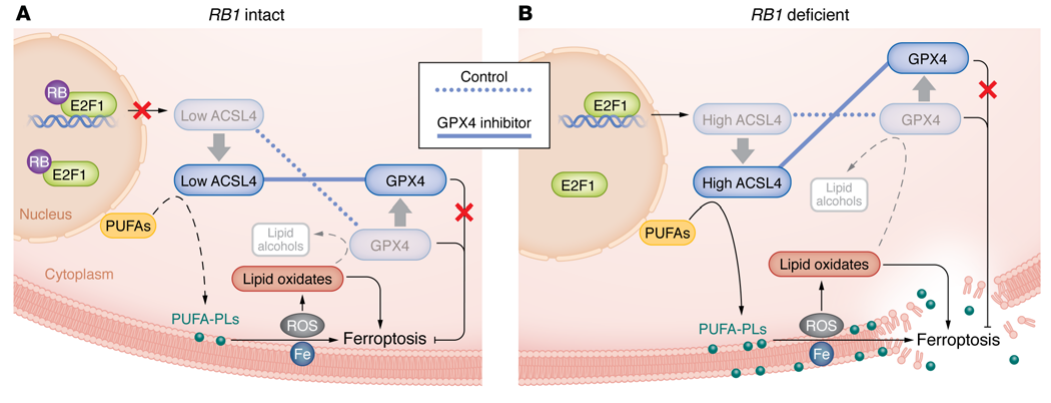
*RB1* loss sensitizes cancer cells to GPX4 inhibitors by tilting the balance between the pro- and antiferroptosis effects of ACSL4 and GPX4, respectively. Ferroptosis is an iron- and ROS-dependent cell death pathway that is tightly controlled by two opposing enzymes, ACSL4 and GPX4. Respectively, ACSL4 and GPX4 induce and deplete lipid peroxidation, a prerequisite for ferroptosis. (**A**) In *RB1*-intact cells, ACSL4 expression is nearly undetectable due to the lack of E2F1-mediated transcriptional activation, resulting in a low level of PUFA-PLs. Due to this weak ferroptotic potential, the cells do not undergo notable ferroptosis, even when GPX4 function is inhibited. (**B**) Upon RB loss, E2F transcription factors induce ACSL4 expression, resulting in PUFA-PL accumulation and a high ferroptotic potential, which is kept in check by GPX4 that is abundantly expressed in most cancer cells and protects the cells from autonomous ferroptosis. However, this strong ferroptotic potential is unblocked when *RB1*-deficient cells are treated with GPX4 inhibitors, resulting in massive ferroptosis and cell death.

Ferroptosis is a recently identified, iron-dependent form of programmed cell death that is distinct from other cell death pathways, such as apoptosis, necrosis, or autophagy (1). During ferroptosis, cells die due to increased lipid peroxidation caused by the accumulation of ROS, leading to cell membrane destruction and, ultimately, cell death (2). Long-chain acyl-CoA synthetase 4 (ACSL4) and glutathione peroxidase 4 (GPX4) are two major enzymes that positively and negatively regulate ferroptosis, respectively (3) (Figure 1A). Like other isozymes of this family, ACSL4 converts free long-chain fatty acids into fatty acyl-CoA esters. However, ACSL4 is unique in that it preferentially utilizes polyunsaturated fatty acids (PUFAs), such as arachidonic acid (AA), as a substrate, facilitating PUFA incorporation into phospholipids on the cellular membranes. Excessive iron-dependent peroxidation of PUFA-containing phospholipids (PUFA-PLs) is a trigger of ferroptosis, implicating ACSL4 as a critical inducer of ferroptotic cell death (4). Contrary to the ferroptosis-inducing role of ACSL4, the phospholipid peroxidase GPX4 is an antioxidant defense enzyme that converts toxic lipid peroxides to nontoxic alcohols, thereby repairing oxidative damage and inhibiting ferroptosis (3, 5).

It has been reported that intracellular iron and its uptake are substantially enhanced in rapidly proliferating cells, such as cancer cells, compared with normal cells (6). In addition, cancer cells often have an abnormal lipid metabolism and increased ROS production (7). All of these predispose cancer cells to iron-dependent, ROS-mediated ferroptosis. Cancer cells counteract this challenge by increasing the expression or activity of ferroptosis-inhibiting pathways, such as GPX4 (8) (Figure 1A). As shown in the Cancer Cell Line Encyclopedia database, almost all prostate cancer (PCa) cell lines tested express GPX4 abundantly and at a level much higher than the benign prostate cell line, PRECLH (https://sites.broadinstitute.org/ccle/). As such, ferroptosis inducers, including GPX4 inhibitors, have some specificity in targeting cancer cells while sparing normal cells. Indeed, several therapeutics for various cancer types have harnessed ferroptosis as a targeted therapy for tumor suppression. For instance, altretamine, a chemotherapy drug that alkylates DNA, causes cell death, and is commonly used for the treatment of late-stage ovarian cancer, has been shown to induce ferroptosis via inhibiting GPX4 (9). Sulfasalazine, a nonsteroidal antiinflammatory drug traditionally used for the treatment of rheumatoid arthritis, has recently been shown to induce ferroptosis in head and neck cancer (10). Further, sorafenib, a multikinase (VEGFR, PDGFR, c-Kit, and RET) inhibitor used to treat hepatocellular carcinoma, can also induce ferroptosis (11).

There is increasing evidence of a correlation between ferroptosis and therapy-refractory or drug-resistant cancer cells (6, 8), such as pancreatic ductal adenocarcinoma (6), small cell lung cancer (12), and triple-negative breast cancer (13). Therapy-resistant PCa cells develop a mesenchymal transcriptional profile that can cause an increased lipid update and synthesis of polyunsaturated lipids (6, 14). Besides lipid peroxidation, iron also plays a vital role in ferroptosis. Thus, the cancer cells adapt by modulating iron metabolism pathways for their advantage. Precisely, PCa cells express higher levels of the transferrin receptors that are important for intracellular iron uptake (15) and reduce the levels of ferroportin, a mediator of iron export, all of which would lead to a higher intracellular iron pool in PCa cells. Moreover, ACSL4 is drastically increased in castration-resistant PCa cells, such as DU145, PC3, and NCI-H660 cells, suggesting enhanced lipid peroxidation. Such an increase in lipids, a labile iron pool, and ACSL4 expression in PCa cells could contribute to increased GPX4 dependency and, therefore, vulnerability to GPX4 inhibitors. Indeed, GPX4 is generally abundantly expressed in PCa cells, which is consistent with its role in preventing the ferroptotic death of cancer cells. Ferroptosis inducers, therefore, may be exploited for cancer therapy both in terms of treating primary PCa and preventing treatment resistance. Zhou et al. have shown that flubendazole, an anthelmintic that is used to treat worm infection, elicits antitumor effects by targeting P53 and promoting ferroptosis in castration-resistant prostate cancer (CRPC) (16). However, the genetic mechanisms that predispose advanced PCa to ferroptosis remain elusive, despite the paramount importance of such understanding for the selection of patient populations for ferroptosis-inducing agents. In this issue of the *JCI*, Wang et al. report that the loss of the retinoblastoma (*RB1*) gene, which occurs in high frequency in many treatment-resistant cancers, including CRPC, sensitizes cancer cells to ferroptosis through upregulation of ACSL4 expression (17).

## RB loss confers a vulnerability to ferroptosis

*RB1* is a tumor suppressor gene that arrests cells in the G_1_/S transition by sequestering E2F family transcription factors, keeping them in a transcriptionally inactive state (18). Accordingly, *RB1* is one of the most frequently mutated and lost genes in various cancers, including PCa. RB loss was reported in approximately 5% of primary PCa cases; this increases to over 30% in metastatic CRPC cases and more than 70% in neuroendocrine PCa (NEPC) cases, which are resistant to the androgen receptor–targeted (AR-targeted) therapies and have very limited treatment options available (19, 20). Interestingly, *RB1* loss is associated with a favorable response to chemotherapy. *RB1*-deficient non–small cell lung cancers, compared with their *RB1*-proficient counterparts, showed efficient and prolonged responses to chemotherapy (12). Similarly, triple-negative breast cancer cases with disrupted RB pathways tend to exhibit a favorable response to neoadjuvant chemotherapy, gamma radiation, and chemotherapy when compared with tumors with proficient RB (21). Even the pancreatic neoplasia with *RB1* loss/K-Ras mutation responds favorably to platinum-based chemotherapy (22). Such increased sensitivity to chemotherapy suggests that *RB1* loss might predispose tumor cells to specific cell death pathways. Indeed, therapy-resistant tumors, many of which harbor *RB1* deficiency, have shown increased vulnerability to ferroptosis inducers (6, 8). However, how RB loss might sensitize tumor cells to ferroptosis has not been characterized to our knowledge.

In this issue of the *JCI*, Wang et al. began their research by treating a panel of PCa cell lines with GPX4 inhibitor RSL3, which is known to induce lipid peroxidation and ferroptosis (Figure 1A) (17). The authors observed ferroptosis in all PCa cell lines tested, consistent with previous studies reporting that cancer cells are predisposed to iron-dependent, ROS-mediated ferroptosis that is kept in check by GPX4 (6). Critically, the authors found that *RB1*-deficient cell lines, such as PC3 and DU145, exhibited much higher sensitivity to GPX4 inhibitor RSL3. This dependency on *RB1* loss was further confirmed using RB knockdown assays in PCa cell lines. Importantly, this finding was not limited to PCa, as RB depletion similarly sensitized lung, liver, and breast cancer cell lines to ferroptosis inducers (17).

Having shown conclusively the role of *RB1* loss in enhancing the ferroptotic potential of cancer cells, the authors took a further step to decipher the underlying molecular mechanisms (17). As RB is well known to inhibit cell growth by suppressing E2F transcriptional activities, the authors focused on ferroptosis regulators that might be target genes of E2F. Analyses of previously published E2F1 ChIP-Seq data sets revealed strong E2F1 protein occupancy at several putative E2F binding motifs at the ACSL4 promoter in various cancer cell lines. Luciferase reporter assays of ACSL4 promoter constructs with various deletion patterns of E2F binding motifs validated that E2F1 binding at the ACSL4 promoter drastically enhances ACSL4 gene transcription. Using RB and/or E2F1 knockdown assays, the authors further demonstrated that RB substantially suppressed ACSL4 gene expression in an E2F1-dependent manner, establishing ACSL4 as a critical downstream target of the RB/E2F pathway. As ACSL4 is an essential enzyme that incorporates PUFAs, such as AA, into phospholipids, a critical step to ferroptosis, the authors showed that RB loss increased lipid peroxidation and ferroptotic potential of cancer cells, which was abolished upon concurrent ACSL4 inhibition. Taken together, these results strongly support a model wherein RB loss increases the basal levels of ACSL4, which leads to PUFA-PL accumulation on the cellular membrane and a strong potential for ferroptosis (Figure 1B). This process is kept in balance by GPX4, which is abundantly expressed in cancer cells, to prevent autonomous ferroptosis. However, the use of GPX4 inhibitors disturbs the equilibrium and unleashes ferroptosis in *RB1*-deficient cells. In contrast, *RB1*-intact PCa cells have nearly undetectable levels of ACSL4, as observed in the Cancer Cell Line Encyclopedia database (https://sites.broadinstitute.org/ccle/), and thus a low basal level of lipid peroxidation. Due to this weak ferroptotic potential, *RB1*-intact cells do not undergo ferroptotic cell death, even when treated with GPX4 inhibitors (Figure 1A). Therefore, by increasing ACSL4 expression and the rate of lipid peroxidation, *RB1* loss raises the extent that cancer cells depend on GPX4 to block ferroptosis and, on the other hand, sensitizes cancer cells to GPX4 inhibitors or, in general, ferroptosis inducers.

## Ferroptosis inducers as potential therapeutics for advanced PCa

De novo NEPC, which originates directly from the neuroendocrine cells of the prostate, is rare and occurs in less than 2% of all prostate cancers (23). However, recent years have seen major increases in treatment-induced NEPC, which transdifferentiates from the luminal/basal cells of the prostate. Cases of treatment-induced NEPCs were reported in 10%–25% of patients with CRPC who have undergone extensive treatment with androgen deprivation therapy and/or AR signaling inhibitors, such as enzalutamide and abiraterone (24). NEPC is an end-stage disease that usually does not respond to standard PCa treatment regimens. These patients have a poor clinical outcome, with a median survival of less than one year (24, 25). Interestingly, one study showed that as high as 90% of NEPC tumors have lost RB protein expression, with 85% of cases harboring *RB1* allelic loss (26). Therefore, the findings by Wang et al. in this issue of the *JCI* that cancers with RB1 loss are hypersensitive to ferroptosis inducers point to a potential therapeutic strategy for the vast majority of patients with NEPC (17).

Wang and authors tested the efficacy of JKE-1674, a recently developed GPX4 inhibitor that is orally active (17, 27), in two mouse models in vivo. First, they showed that JKE-1674 markedly reduced the growth of RB-knockdown PC3 xenograft tumors, attributed to increased ferroptosis. It is somewhat disappointing that JKE-1674 failed to inhibit the control PC3 xenografts, despite low RB in these cells. It will be helpful to test additional RB1 mutant models, such as DU145 cells, to provide further information on the actual therapeutic potential of JKE-1674 in cells with variable RB1 levels. Importantly, the authors also tested JKE-1674 in the *Pten/Rb1* double-knockout mice with metastatic NEPC tumors in an immunocompetent setting. JKE-1674 drastically reduced the growth of the primary tumors and prevented their metastasis to the lungs, liver, and lymph nodes, and the mice showed an increase in overall survival. These results strongly support that ferroptosis inducers like JKE-1674 may be useful in treating patients with NEPC to inhibit tumor growth, reduce metastasis, and extend patient life or promote longevity (17).

## Conclusions and future directions

Drug resistance in cancer treatment represents a major clinical challenge and a leading cause of cancer-associated deaths. FDA approval and clinical use of high-affinity AR pathway inhibitors, such as enzalutamide, abiraterone, and apalutamide, in the past decade, have greatly improved care for patients with PCa and substantially prolonged patients’ lives (28). As such, there are notable increases of treatment-induced NEPC cases, most of which harbor *RB1* loss and lack effective treatment modalities. The discoveries made by Wang et al. (17) in this issue of the *JCI* are thus highly important, as they provide a potential targeted therapy for these otherwise untreatable forms of cancers. Furthermore, these findings provide a molecular mechanism explaining the correlation between ferroptosis and treatment-refractory cancer, wherein *RB1* is often lost. The study highlights ferroptosis as a targeted therapy in advanced cancer and warrants the development of ferroptosis inducers for clinical use. It identifies JKE-1674 as an orally available ferroptosis inducer that causes no obvious toxicity in the mice and inhibits tumor growth and metastasis in vivo. Additional animal experiments are required to test the efficacy of the drug in additional mouse models, including patient-derived xenografts of varying *RB1* status and/or RB expression levels. Further pharmacokinetics, biosafety profiling, and dosing schema of JKE-1674 will need to be characterized in the mice and in humans in phase I clinical trials. On the other hand, patient selection using appropriate biomarkers will likely improve the success rates of future clinical trials involving ferroptosis inducers. Genetic and immunohistochemistry assays could be developed to evaluate RB1 allelic deletion and RB1 protein loss, respectively. Analogously, it may be useful to directly measure ACSL4 levels and evaluate ACSL4 as a biomarker for patient selection in clinical trials. Finally, due to the critical role of ACSL4 expression in sensitizing tumors to ferroptosis inducers, it is intriguing to determine whether ACSL4 is epigenetically silenced in PCa and whether epigenetic inhibitors may be exploited for combination therapy with ferroptosis inducers to benefit a broader patient population.

## Version 1 05/15/2023

Electronic publication
